# Gynecologic infection rates after ablation treatment for cervical intraepithelial neoplasia grade 2 and higher (CIN2+): Secondary analysis of a non-inferiority randomized trial

**DOI:** 10.1371/journal.pgph.0003333

**Published:** 2024-07-10

**Authors:** Rachel Masch, Gabriel Conzuelo-Rodriguez, Jameson A. Mitchell, Karla Alfaro, Montserrat Soler, Luis F. Chavez, Suhui Wu, Jinfen Sun, Longhua Hu, Dayana Marinela-Hernandez, Todd A. Alonzo, Juan C. Felix, Miriam L. Cremer

**Affiliations:** 1 Department of Obstetrics, Gynecology and Reproductive Science, The Mount Sinai Hospital, New York, New York, United States of America; 2 Basic Health International, Pittsburgh, Pennsylvania, United States of America; 3 Cleveland Clinic Lerner College of Medicine, Cleveland, Ohio, United States of America; 4 Obstetrics, Gynecology Institute, Cleveland Clinic, Cleveland, Ohio, United States of America; 5 Department of Obstetrics and Gynecology, Shanxi Bethune Hospital, Shanxi Academy of Medical Sciences, Tongji Shanxi Hospital, Third Hospital of Shanxi Medical University, Taiyuan, Shanxi Province, China; 6 Department of Obstetrics and Gynecology, Tongji Hospital, Tongji Medical College, Huazhong University of Science and Technology, Wuhan, Hubei Province, China; 7 Hospital Primero de Mayo, Instituto Salvadoreño del Seguro Social, San Salvador, El Salvador; 8 Department of Population and Public Health Sciences, Keck School of Medicine, University of Southern California, Los Angeles, California, United States of America; 9 Department of Pathology, Medical College of Wisconsin, Milwaukee, Wisconsin, United States of America; PLOS: Public Library of Science, UNITED STATES

## Abstract

Although concerns have been raised regarding potential infection and morbidity in women undergoing ablation treatment for cervical precancer in low- and middle-income countries (LMIC), there is extremely limited data to substantiate this claim. This is a secondary analysis of a randomized non-inferiority trial (id: NCT03084081) that compares the efficacy and safety of three ablation treatments for biopsy-confirmed cervical intraepithelial neoplasia grade 2 or higher (CIN2+): CO_2_ gas-based cryotherapy, non-gas cryotherapy, and thermal ablation (TA). Here, we present findings regarding the incidence of sexually transmitted infections (STI) and vaginitis post-treatment. Samples were collected at enrollment and again at 6 weeks post-treatment and assessed for STIs (*Chlamydia trachomatis* (CT), *Neisseria gonorrhea* (NG), and *Trichomonas vaginalis* (TV)) and vaginitis (Bacterial vaginosis (BV) and/or *Candida albicans* (Candida)). This analysis reflects 864 women with baseline and 6-week follow-up data. None of the ablative treatments put women at increased risk for STIs (CT, NG, TV) or vaginitis (BV, Candida). While most women adhered to post-treatment recommendations (97%) and no difference by treatment arm was observed, the incidence of STIs at follow-up in women that did not adhere with a given recommendation was higher compared to their adherent counterparts. The incidence of gynecologic infection did not increase with any of the ablation treatments from baseline to the six-week follow-up. Non-gas cryotherapy and TA emerge as safe alternatives to gas-based cryotherapy with respect to gynecologic infection rates.

## Introduction

Cervical cancer is preventable but remains a leading cause of cancer in women globally, with approximately 90% of incident cases occurring in low- and middle-income countries (LMIC) [1]. In 2020, an estimated 604,000 women were diagnosed with cervical cancer worldwide and approximately 342,000 women died from the disease [2]. High risk human papilloma virus (hrHPV) is the cause of almost all cervical cancers. While there is a vaccine to prevent several subtypes of hrHPV, secondary prevention of cervical cancer through routine screening and treatment of high-grade precancerous disease, hereinafter referred to as cervical intraepithelial neoplasia 2 or higher (CIN2+), remains necessary for the foreseeable future [3].

Cryotherapy, the standard of care for CIN2+ treatment in LMIC, has been shown to be effective with few side effects and little impact on fertility [4]. However, implementation of this technology in LMIC is limited because of difficulties in gas supply and transport, expense, and maintenance [5,6]. Two dual electric/battery powered alternative technologies—the non-gas based cryotherapy (CryoPen), and the thermal ablation (TA) device—are smaller, portable treatment modalities that do not require an expensive consumable to function.

Although the World Health Organization (WHO) guidelines [7] endorsed cryotherapy and TA (i.e., ablative therapies) as viable CIN2+ treatment methods, the committee noted a paucity of data on risk of infection after treatment and was unable to provide specific recommendations on the use of prophylactic antibiotics. Studies that have evaluated infection after ablative therapies report a 1–3% risk of infection [8–10]. However, these studies assessed infection only by inspection or by questionnaires rather than by laboratory assessment, which reduced the validity of the findings. In fact, there is limited data on gynecologic infection after ablative therapy for CIN2+ using objective measurements of infection.

The aim of this study is to present findings on the incidence of laboratory-confirmed sexually transmitted infections (STIs)–*Chlamydia trachomatis* (CT), *Neisseria gonorrhoeae* (NG), and *Trichomonas vaginalis* (TV)—as well as vaginal overgrowth (Bacterial vaginosis [BV] and *Candida albicans* [Candida]) in the context of a randomized clinical trial comparing three different ablation therapies for CIN2+ treatment (NCT03084081).

## Methods

### Ethics statement

The protocols of this study were evaluated and approved by the Cleveland Clinic institutional review board (IRB), as well as the ethics committee at each participating site: Consejo Superior de Salud Publica (El Salvador); Comite de Investigaciones y Etica Institucional, Facultad de Medicina Pontificia Universidad Javeriana ‐ Hospital Universitario San Ignacio (Colombia); and Shanxi Academy of Medical Science Shanxi Grand Hospital Medical Ethics Committee (China). All participants provided written or oral informed consent. A data safety monitoring board periodically reviewed enrollment and preliminary study results.

### Study population

**Fig 1:**
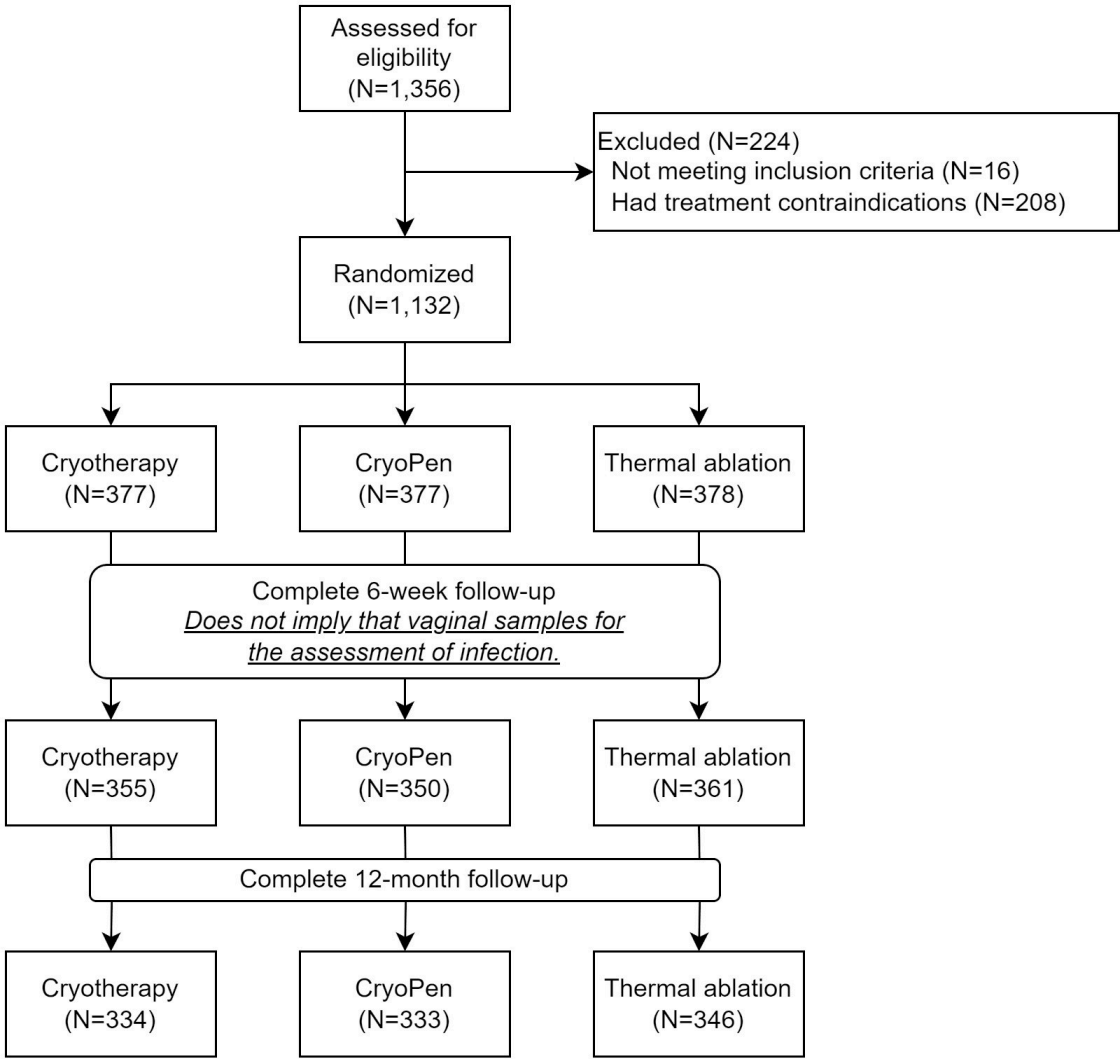
Study design and enrollment.

Patients were recruited from the Hospital 1° de Mayo of the Instituto del Seguro Social in San Salvador, El Salvador; Hospital Universitario San Ignacio in Bogotá, Colombia; the Shanxi Bethune Hospital in Taiyuan (Shanxi Province), and in Xinxiang (a semi-rural area in Henan Province), China. A total of 1,132 women across all sites were randomized to treatment from June 19, 2017, to July 8, 2022 (Fig 1). During the initial visit, women were considered eligible if they were over 18 years of age, had biopsy confirmed CIN2+, consented to participate in the study, and provided a reliable address. Women with a history of destructive surgery of the cervix in the last 5 years, history of a total hysterectomy, or who were pregnant or planning to become pregnant during the study timeframe were excluded.

Eligible patients provided demographic and medical history details and underwent pelvic examination by a trained gynecologist to evaluate eligibility for ablation treatment. To be eligible for ablation the lesion covered less than 75% of the cervix, did not extend into the endocervix or onto the vagina, and did not appear to be frank cancer. Women without contraindications were assigned to a treatment arm using a computerized randomization module in REDCap, a HIPAA compliant data management and storage system [11,12]. The study PI and local PIs were blinded for the main study outcomes until follow-up was completed. Vaginal samples were collected by the provider during the initial pelvic exam to test for STIs (*CT*, *NG*, *TV*) as well as vaginitis (BV, *Candida*). This process was repeated by a provider at the 6-week follow-up visit. Additional details on the study protocol can be found in the supplementary materials.

### Analytical sample

A total of 1,066 women that attended their 6-week visit were initially considered for this analysis (El Salvador = 254; Colombia = 67; China = 745). However, cost prohibitions and logistical difficulties due to COVID-19 pandemic precluded 135 samples from El Salvador and all samples from Colombia from being analyzed. Therefore, the final analysis was limited to 864 women whose vaginal samples were analyzed for STIs (El Salvador = 119; China = 745).

### Specimen collection and laboratory process

Once randomized, participants underwent speculum examination. A swab was introduced into the vagina by a clinician and fully rotated 3–5 times to collect the sample for analysis of *CT*, *NG*, *TV*, BV, and *Candida*. If an infection was clinically suspected the patient was referred for further evaluation and their ablation treatment was rescheduled (N = 7). If no infection was suspected, the patient was treated by their randomly assigned ablation method. Patients were seen six weeks after treatment to assess infection status, adherence to post-treatment recommendations, and treatment side effects.

In El Salvador, the samples were stored at room temperature and then sent to the Medical College of Wisconsin (MCW) for analysis. At MCW, *CT/NG* testing was done using the Roche Cobas *CT/NG* test. BV and *TV* analyses were performed using the BD MAX Vaginal Panel, which also reports several Candida species. In China, human tissue samples are not allowed to be sent abroad. Therefore, these samples were analyzed by a local Chinese laboratory using CT/NG/UU real-time polymerase chain reaction (PCR) Kit (Ref. No.: HBRT-STD3) manufactured by Chaozhou Hybribio Biochemistry Limited (Guangdong, PR China) with National Medical Products Administration (NMPA) certification to detect CT and NG. PCR and gel electrophoresis were used to analyze BV, *TV*, and *Candida*. Of note, the panel for BV in China reported the presence of any strain of *Gardnerella vaginalis*, many of which are considered normal flora.

### Treatment

#### CO_2_-based cryotherapy procedure

The cryotherapy probe tip was placed only on the cervix (no vaginal contact) to ensure the entire lesion was covered. Cryotherapy was applied for 3 minutes of freeze, 5 minutes of thaw, and another 3 minutes of freeze. The cryotip was then removed by gently rotating it when complete thawing had occurred. A MedGyn cryotherapy (Medgyn Products, Illinois, USA) unit with a 19-20mm conical tip was used with a 27 kg tank of medical grade CO_2_.

#### The non-gas based cryotherapy (CryoPen) procedure

**Fig 2:**
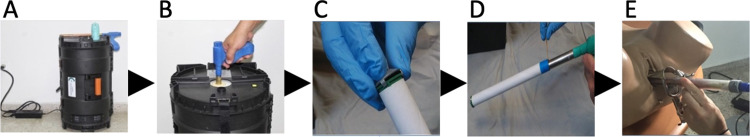
Non-gas based cryotherapy (CryoPen) equipment.

The CryoPen (Fig 2) unit was plugged into an electrical outlet and was ready when a temperature indicator light turned green. A 19mm conical aluminum probe tip was placed onto a cardboard applicator, and this was placed on the cervix with the nipple of the probe tip in the external os. The core handle was then inserted into the cardboard applicator, stabilized, and a single 5-minute freeze was applied. After the treatment, the core handle was removed, and with complete thawing, the probe tip and cardboard applicator were extracted by gently rotating the tip.

#### Thermal ablation

**Fig 3:**
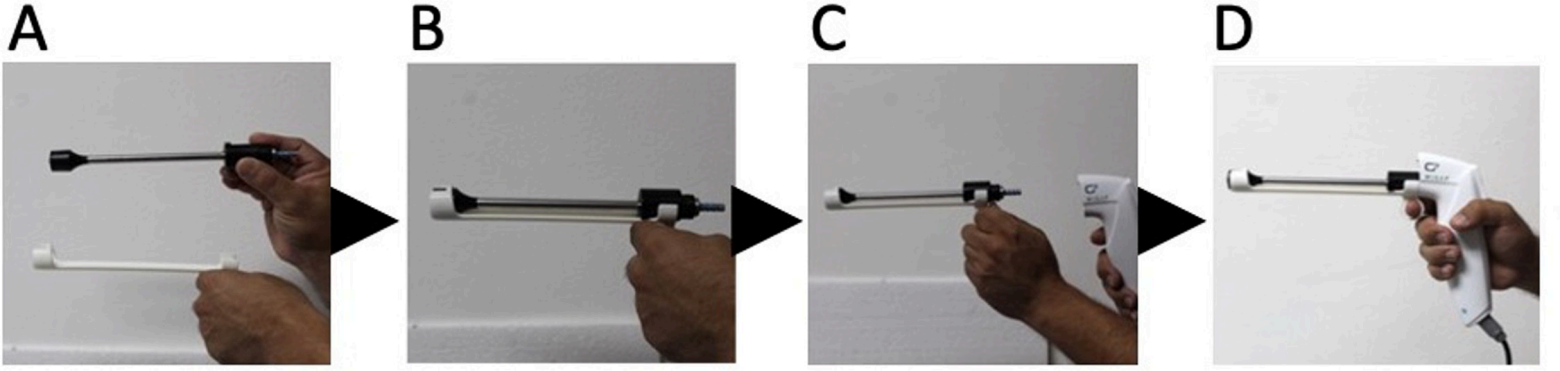
WiSAP thermal ablation equipment.

The WiSAP (Brunnthal, Germany) Thermal Ablator (Fig 3) uses electricity or a battery for its power source. The probe tip was heated to 100°C and placed onto the cervix with the nipple in the external os for 40 seconds. Additional 20-second applications were used to ensure ablation of the entire transformation zone.

### Statistical analysis

Central tendency measures and percentages were used to describe demographic and clinical characteristics. Differences between countries were calculated using one-way ANOVA or Wilcoxon Rank Sum test for continuous variables, and Chi-square test or Fisher’s exact test for discrete variables. The incidence of any STI at 6-week visit was calculated in women that had no evidence of infection in their initial vaginal sample. To account for differences in BV reporting between China and El Salvador (i.e., any *G*. *vaginalis* was reported as BV in China), a sensitivity analysis was performed in which women who were only positive for BV in China were not considered as having an infection. In both scenarios, differences in STI incidence by treatment arm were assessed. Finally, the infection status at follow-up visit by adherence to post-treatment recommendations was evaluated. All statistical analyses were performed using R (R Foundation for Statistical Computing, Vienna, Austria).

## Results

A total of 864 women were included in this analysis. Approximately 86% of whom were recruited in China. The average age of participants in China was 37 years (SD = 9), with over 70% reporting having at least a high school education, and 57% reporting only one lifetime sexual partner. In contrast, women from El Salvador were younger (mean age of 35 years (SD = 9)), had less years of education (14% with high school completion), and had more lifetime sexual partners (only 18% reported only one-lifetime partner). While women in El Salvador reported living in urban environments more often than their Chinese counterparts (89% vs 75%), their mean commuting time from home to the clinic was longer (80 vs. 59 minutes). Additional details can be found in Table 1.

**Table 1:** Demographic characteristics.

Characteristic	Overall N = 864	El Salvador N = 119	China N = 745	p-value^3^
Age^1^	37 (9)	35 (9)	37 (9)	<0.01
Education^2^				<0.01
Less than high school	181 (42%)	102 (86%)	79 (25%)	
High school complete	94 (22%)	17 (14%)	77 (25%)	
College or above	156 (36%)	0	156 (50%)	
(Missing)	433	0	433	
No. sexual partners^2^				<0.01
One	447 (52%)	22 (18%)	425 (57%)	
Two	221 (26%)	39 (33%)	182 (24%)	
Three or more	196 (23%)	58 (49%)	138 (19%)	
Smoking^2^				<0.01
Never	818 (95%)	97 (82%)	721 (97%)	
Former	24 (2.8%)	17 (14%)	7 (0.9%)	
Current	22 (2.5%)	5 (4.2%)	17 (2.3%)	
Area of residence^2^				<0.01
Urban	668 (77%)	16 (89%)	562 (75.4%)	
Rural	196 (23%)	13 (11%)	183 (25%)	
Commuting time to clinic (mins) ^1^	62.6 (62.2)	82.3 (48.0)	59.4 (63.6)	<0.01

^1^Mean (SD).

^2^n (%).

^3^ Wilcoxon rank sum test; Pearson’s Chi-squared test; Fisher’s exact test.

During the initial visit, 7.4% of enrolled patients tested positive for STI (*NG*, *CT*, *TV*), with 9.3% and 7.2% in El Salvador and China, respectively. The most common STI was *TV*, affecting 3.7% of the study sample. When examining the prevalence of vaginitis, we found that 73% of patients tested positive for BV with 23% and 81% in El Salvador and China, respectively. However, as noted earlier, China reported the presence of any *G*. *vaginalis* in their results, thus increasing the likelihood of testing positive for BV infection. Candida was identified in 5% of patients; the prevalence of *Candida* was higher in El Salvador (12%) than in China (4%) (Table 2).

**Table 2:** Baseline prevalence of STIs and vaginal overgrowth by site.

Organism^1^	Overall N = 864	El Salvador N = 119	China N = 745
Any STI	54 (7.4%)	5 (9.3%)	49 (7.2%)
*N*. *gonorrhea*	3 (0.3%)	0	3 (0.4%)
*C*. *trachomatis*	25 (2.9%)	3 (2.5%)	22 (3.0%)
*T*. *vaginalis*	27 (3.7%)	2 (3.8%)	25 (3.7%)
Bacterial Vaginosis^2^	633 (73%)	27 (23%)	606 (81%)
*C*. *albicans*	43 (5.0%)	14 (12%)	29 (3.9%)

Note: Participants could test positive for multiple infections.

Sexually Transmitted Infection (STI).

^1^ N (%).

^2^ In China, includes women with any strain of *G*. *vaginalis*.

A total of 147/864 (17.0%) women had no evidence of STI or vaginitis in their vaginal sample from the initial visit (Table 3). In these women, the overall incidence of STI following treatment was 1% and was similar across treatment (p = >0.9). With respect to overgrowth, 44% and 6% of patients had BV and *Candida* identified at the 6-week follow-up visit, respectively. Like STIs, the incidence of overgrowth did not differ by treatment arm (Table 3). In the sensitivity analysis, in which women from China who tested positive exclusively for BV were considered as uninfected, a total of 633/864 (73%) had no evidence of infection at baseline. Like the main analysis, the overall incidence of STI was low (1.9%) and did not vary by treatment arm. Details on the sensitivity analysis are presented in the supplemental materials (S1 Table).

**Table 3:** Incidence of STIs and vaginal overgrowth in women without pathogens reported in their initial vaginal sample, by treatment arm.

Organism^1^	Overall N = 206	CO_2_ N = 72	Non-gas based cryotherapy (CryoPen) N = 61	TA N = 73	p-value^2^
Any STI	1 (0.7%)	0	0	1 (2.0%)	>0.9
*N*. *gonorrhea*	0	0	0	0	
*C*. *trachomatis*	0	0	0	0	
*T*. *vaginalis*	1 (0.7%)	0	0	1 (2.0%)	>0.9
Bacterial Vaginosis^3^	69 (36%)	23 (35%)	24 (42%)	22 (32%)	0.5
*C*. *albicans*	11 (5.8%)	5 (7.8%)	1 (1.8%)	5 (7.2%)	0.3

Note: Participants could test positive for multiple infections

Sexually Transmitted Infection (STI); Thermal Ablation (TA).

^1^n (%).

^2^Pearson’s Chi-squared test; Fisher’s exact test.

^3^In China, includes women with any strain of *G*. *vaginalis*.

While most women adhered to post-treatment recommendations (97%) and no difference by treatment arm was observed [13], the incidence of STIs at follow-up in women that did not adhere with a given post-treatment recommendation was larger compared to their adherent counterparts. For example, compared to women that followed the recommendation of avoiding sexual intercourse, those that did not were more likely to test positive for at least one infection at follow-up, 73.8% vs. 83.3%. A similar proportion was found for the recommendation of avoiding vaginal douching, 74.1% vs. 88.9% (Table 4).

**Table 4:** Presence of *any* STI or overgrowth at 6-week follow-up by adherence to post-treatment recommendations.

Recommendation^1^	Overall N = 746	Infection at 6-week follow-up		p-value^2^
		No N = 205	Yes N = 541	
Avoid entering the pool/sea				0.1
Did not adhere	6	3 (50.0%)	3 (50.0%)	
Adhere	664	136 (20.5%)	528 (79.5%)	
(Missing)	76			
Avoid sexual intercourse				0.3
Did not adhere	42	6 (14.3%)	36 (85.7%)	
Adhere	628	133 (21.2%)	495 (78.8%)	
(Missing)	76			
Avoid performing vaginal douche				0.7
Did not adhere	9	1 (11.1%)	8 (88.9%)	
Adhere	661	138 (20.9%)	523 (79.1%)	
(Missing)	76			
Avoid using tampons				>0.9
Did not adhere	1	0 (0.0%)	1 (100.0%)	
Adhere	665	138 (20.8%)	527 (79.2%)	
(Missing)	80			

Notes: Missing values in recording of adherence are due to a later inclusion of these questions in the study.

^1^n (%); row percentages.

^2^Fisher’s exact test; Pearson’s Chi-squared test.

## Discussion

### Summary of main results

Findings from this study indicate that treatment of CIN2+ lesions with CO2-cryotherapy, non-gas based cryotherapy (CryoPen), or TA is neither associated with an increase in gynecologic infection (STI or vaginitis) nor does it put women at higher risk of developing an infection. Overall, infection rate at the 6-week follow-up was low for all organisms. Of note, there was a high baseline prevalence and incidence of BV among women from the China sites, but the assay that was used reported on the presence of any strain of *G*. *vaginalis*, which is often found in the normal vaginal flora. Indeed, up to 87% of women in one study were found to have *G*. *vaginalis* without having pathogenic BV [14]. When BV was excluded from analysis, overall baseline prevalence of infection was 10.8%. Regardless, the most significant finding is that using the same assay for both pre- and post-treatment infection evaluation, there was no evidence to support a difference in gynecologic infection rates from the initial to the 6-week visit for any of the treatments. Additionally, although adherence to post-treatment recommendations was high, infection rates at follow-up were higher in women that did not follow them.

### Results in the context of published literature

Our results align with previously published data on infection following ablative therapies. Singh et.al [15]. reported only 2 infections out of 158 (1.3%) patients treated with cryotherapy (N = 68) or thermal ablation (N = 90), one in each arm. Similarly, Mitchell et.al. [8], observed only 1 infection among 139 (0.7%) patients treated with cryotherapy. Finally, Duan et.al. [9], found zero infections in 145 women treated with either cryotherapy (N = 71) or thermal ablation (N = 74). Unlike these studies, our approach used an objective laboratory assessment of infection rather than clinical evaluation or the reporting of symptoms, which strengthens the evidence that none of the ablative therapies confers an inherent risk of infection. Our findings also support the decision to not treat women with prophylactic antibiotics at the time of treatment.

### Strengths and weaknesses

Treatment randomization mitigated the confounding bias in our study. Additionally, result stratification by country does not show an association between treatment and gynecologic infection, thus increasing the external validity of these results. Finally, the risk of misclassification error was decreased by using standardized procedures to test all women for gynecologic infection during both visits, regardless of whether they were experiencing symptoms.

Since different laboratories were used to evaluate the samples from China and El Salvador, the assays used at each site had different thresholds for being positive and negative, thus limiting our capacity to compare infection rate across sites. Nevertheless, the assays were standardized at each site (i.e., the same lab and procedures were used for each of the patients’ visits at baseline and 6-week follow-up in each country), which allows for pre- and post- comparisons and reduces the risk of bias. The high prevalence of BV in China is explained by their inclusion of any strain of *G*. *vaginalis*, many of which are components of the normal vaginal flora and not necessarily pathogenic. Despite this high rate, ***the important finding from this study is that none of the ablation devices increased infection when compared to the others***. Lastly, HIV status was not evaluated in this study and these results may not be generalizable to this population.

### Implications for practice and future research

This study further supports the idea that treatment of CIN2+ lesions with non-gas based cryotherapy (CryoPen) and TA do not increase the risk of gynecologic infection in non-HIV populations. In fact, none of the ablative treatments in this study put women at increased risk for infection 6-weeks after their procedure. Furthermore, it adds to the growing body of literature on the safety of TA and CyoPen, which overcome limitations inherent to CO_2_-cryotherapy. Future research should evaluate whether our findings apply to a population of women living with HIV.

## Supporting information

S1 Checklist Inclusivity in global research. (DOCX)

S2 Checklist (DOC)

S1 Table Incidence of STIs and vaginal overgrowth in women without pathogens reported in their initial vaginal sample, by treatment arm (sensitivity analysis). (DOCX)

S1 Protocol Research protocol. (DOCX)

S1 Codebook (XLSX)

S1 Data (CSV)
