# Supplementary material for: Gynecologic infection rates after ablation treatment for cervical intraepithelial neoplasia grade 2 and higher (CIN2+): Secondary analysis of a non-inferiority randomized trial
Source: PLOS Glob Public Health. 2024 Jul 10;4(7):e0003333. doi: 10.1371/journal.pgph.0003333 (PMC11236093; doi:10.1371/journal.pgph.0003333)
Supplement: S1 Table — (DOCX) [file pgph.0003333.s003.docx]

**Supplemental information**

**S1 Table. Incidence of STIs and vaginal overgrowth in women without pathogens reported in their initial vaginal sample, by treatment arm (sensitivity analysis^1^).**

| Organism^2^ | Overall  N = 692 | CO_2_  N = 226 | CryoPen^®^  N = 229 | TA  N = 237 | p-value^3^ |
| --- | --- | --- | --- | --- | --- |
| Any STI | 12 (1.9%) | 2 (1.0%) | 4 (1.9%) | 6 (2.8%) | 0.4 |
| *N. gonorrhea* | 2 (0.3%) | 1 (0.5%) | 1 (0.4%) | 0 (0%) | 0.5 |
| *C. trachomatis* | 3 (0.4%) | 0 (0%) | 0 (0%) | 3 (1.3%) | 0.11 |
| *T. vaginalis* | 7 (1.1%) | 1 (0.5%) | 3 (1.4%) | 3 (1.4%) | 0.7 |
| Bacterial Vaginosis | 480 (71%) | 147 (67%) | 167 (75%) | 166 (72%) | 0.3 |
| *C. albicans* | 22 (3.3%) | 7 (3.2%) | 4 (1.8%) | 11 (4.7%) | 0.2 |
| Note: Participants could test positive for multiple infections  Sexually Transmitted Infection (STI); Thermal Ablation (TA)  ^1^ Women from China who exclusively tested positive for BV are considered as uninfected.  ^2^ N (%)  ^3^ Pearson's Chi-square test; Fisher's exact test | | | | | |
