# Supplementary material for: Gynecologic infection rates after ablation treatment for cervical intraepithelial neoplasia grade 2 and higher (CIN2+): Secondary analysis of a non-inferiority randomized trial
Source: PLOS Glob Public Health. 2024 Jul 10;4(7):e0003333. doi: 10.1371/journal.pgph.0003333 (PMC11236093; doi:10.1371/journal.pgph.0003333)
Supplement: S1 Protocol — (DOCX) [file pgph.0003333.s004.docx]

Research Protocol

A randomized controlled trial comparing cure rates of cervical intraepithelial neoplasia grade 2 and higher (CIN2+) treated with CO_2_-based cryotherapy, CryoPen, and thermoablation

Sponsor: National Cancer Institute- UH3CA189883

Table of Contents

[List of Abbreviations](#h.30j0zll)

[1.0](#h.1fob9te)  [Background and rationale](#h.1fob9te)

[2.0](#h.3znysh7)  [Study objectives](#h.3znysh7)

[3.0](#h.2et92p0)  [Study design and statistical analysis](#h.2et92p0)

[3.1](#h.tyjcwt)  [Study design](#h.tyjcwt)

[3.2](#h.1t3h5sf)  [Sample size and statistical analyses](#h.1t3h5sf)

[4.0](#h.4d34og8)  [Research participants](#h.4d34og8)

[4.1](#h.2s8eyo1)  [Characteristics of research participants](#h.2s8eyo1)

[4.2](#h.17dp8vu)  [Inclusion and exclusion criteria](#h.17dp8vu)

[5.0](#h.3rdcrjn)  [Study procedures](#h.3rdcrjn)

5.1 Initial Exam

5.2 [Study procedures for cervical ablations](#h.26in1rg)

5.3 [Laboratory evaluation](#h.lnxbz9)

[6.0](#h.35nkun2)  [Recruitment](#h.35nkun2)

[7.0](#h.1ksv4uv)  [Consent process](#h.1ksv4uv)

8.0 [Study instruments](#h.44sinio)

[9.0](#h.2jxsxqh)  [Risks](#h.2jxsxqh)

[10.0 Mitigation of risks](#h.z337ya)

[11.0 Benefits](#h.3j2qqm3)

[12.0 Study and safety monitoring](#h.1y810tw)

[13.0 Managing and reporting adverse events and unanticipated problems](#h.4i7ojhp)

[13.1 Adverse Events](#h.2xcytpi)

[13.2 Serious Adverse Events](#h.1ci93xb)

[13.3 Reporting procedures for AEs, SAEs, and unanticipated problems](#h.3whwml4)

[14.0 Confidentiality and data management](#h.2bn6wsx)

[15.0 Study costs](#h.qsh70q)

[16.0 Care for injury](#h.3as4poj)

[17.0 Compensation](#h.1pxezwc)

[18.0 Investigator responsibilities](#h.49x2ik5)

[Appendices](#h.2p2csry)

### List of Abbreviations

AE Adverse Event

C Celsius

FDA Food and Drug Administration, United States

HPV Human papillomavirus

IRB Institutional Review Board

kg kilograms

mm millimeters

PI Principal Investigator

PID Pelvic inflammatory disease

REC Research Ethics Committee

SAE Serious Adverse Event

SCJ Squamo-Columnar Junction

# 1.0 Background and rationale

Cancer of the cervix is preventable but continues to cause the deaths of an estimated 275,000 women worldwide each year, more than 88 percent of them in developing countries.^1^ Vaccination against human papillomavirus (HPV), a means of primary prevention, has been shown to be over 90 percent effective in protecting against the lesions that lead to the majority of cervical cancer cases, but only if administered before viral infection,^2,3^ which occurs soon after initiation of sexual activity.^4^ Secondary prevention is accomplished with cervical screening followed by treatment of precancer and is essential for protecting women who have been sexually active. The World Health Organization recently declared that “screening for cervical cancer once, at age 40, followed by removal of any discerned precancerous lesion” is a “best buy.”^5^ Best buys are defined as actions that should be undertaken immediately to produce accelerated results in terms of lives saved, diseases prevented, and heavy costs avoided.

Screening, with treatment when necessary, has been the key to the 70 percent or more reduction in cervical cancer cases in industrialized countries over the past 50 years, and it is critical to reducing the huge burden of cervical cancer in low-resource countries. Screening technologies appropriate to low-resource settings have advanced considerably in the past decade, but a key component of any effective screening strategy is treatment—and treatment technologies have lagged behind. Treatment methods for developing countries must be low-cost, effective, and adaptable for areas with limited resources in terms of infrastructure and health care providers.

Cryotherapy, the current gold standard for treatment of precancerous cervical lesions in low resource settings,^6-9^ has many appealing features; however, in recent years a number of issues—particularly the use of compressed gas, which requires storage and replenishment of large and potentially dangerous tanks—have emerged as clear limitations.

Two technologies—the CryoPen^®,^ manufactured and sold by CryoPen^®^, Inc. (TX, USA), and the cold coagulator commercialized by WISAP Medical Technology (Germany) are treatment options for precancerous cervical lesions that run on electricity and avoid the difficulties associated with compressed gas. The CryoPen^®^ is a treatment option for precancerous cervical lesions that are able to meet core criteria of cost, safety, and potential for use in remote campaign locations as well as in clinic settings. The CryoPen^®^ differs from previous cryotherapy methods in that it does not require an external source of gas (a tank), since the gas for cooling is built into the system. The original device has been redesigned to be portable, durable, low-cost and low-maintenance for use in low- and middle-income countries (LMICs).

Cold coagulation (“thermocoagulation”) has been used since the 1970s for treatment of cervical lesions. In spite of its name, this is treatment with a probe heated to temperatures of 100 to 120ºC. Henceforth this treatment method will be referred to as “thermoablation.” It is also an effective, inexpensive method that does not require high-level healthcare providers.

The purpose of this study is to conduct a randomized non-inferiority clinical trial to compare the LMIC-adapted CryoPen^®^ and WISAP C3 thermoablator to CO_2_-based cryotherapy for the treatment of CIN2 or more severe diagnoses (“CIN2+”) but excluding microinvasive or invasive cervical cancer.

# 2.0 Study objectives

**The primary objective of the study** is to compare CIN2+ cure rates at twelve-months post-treatment with the LMIC-adapted CryoPen^®^, the WISAP thermoablator, and CO_2_-based cryotherapy.

**Secondary objectives of the study are:**

- To measure patient acceptability of the CryoPen^®^ and thermoablator with pain scores and surveys
- To actively assess side effects of cryotherapy and thermoablation including infection, pain, bleeding, discharge, and stenosis at a six-week post-treatment visit
- To evaluate the sensitivity of high-risk HPV DNA testing post-ablation

# 3.0 Study design and statistical analysis

### 3.1 Study design

Women will be recruited from the Salvadorian Social Security Hospital (ISSS) in San Salvador, El Salvador, La Liga Contra el Cáncer in Lima, Peru, Hospital Universitario San Ignacio (HUSI) and Ginesalud SAS in Bogota, Colombia and Shanxi Dayi Hospital (SDH) in Taiyuan, China. Women attending a regional screening program will also be recruited at three hospitals in Xinxiang, Henan Province, China: Yanjin County Women and Children Hospital, Fengqiu County Women and Children Hospital, and Weihui Municipal Hospital for Women and Children Healthcare.

All hospitals are referral sites for women with abnormal cytology.

Eligible women will be those over the age of 18 with biopsy results of CIN2+ (high grade CIN not otherwise specified) who consent to participate after being informed about the study and are willing and able to provide a permanent and reliable address. Women will be excluded if they are pregnant or plan to become pregnant during the study, have had previous surgery destructive to the cervix in the last 5 years, or have a history of total hysterectomy.

Women who meet the eligibility criteria listed above, will be invited to participate in the study. The diagnosis of CIN2+ will be based on the local pathology diagnosis.

Women will be randomly assigned to one of three study arms:

1. CO_2_-based cryotherapy (double freeze)
2. LMIC-adapted CryoPen® (single freeze)
3. Thermoablation for 40 seconds at 100^º^C followed by additional 20-second applications if necessary according to clinician’s criteria in order to fully ablate the squamo-columnar junction (SCJ).

Recruitment, enrollment, and patient flow are summarized in **Figure 1**.

Figure 1 Consort diagram of study patient flow

The single or double freeze modalities were chosen given the results of the UH2 trial where single-freeze CryoPen® achieved a greater depth of necrosis than double freeze, and the WHO cryotherapy guidelines which endorse CO_2_ double-freeze as the standard cryotherapy treatment. While these are the preferred modalities, in a few cases clinicians may decide to repeat the CryoPen^®^ modality to correct placement of the first application and maximize treatment efficacy and patient safety.

We will approach approximately 1,602 women with CIN2+ during a 2.5 year enrollment phase (30 months). Assuming an approximately 80% participation rate,^10^ approximately 1,281 women will be enrolled— 1,152 (90%) will be eligible for cryotherapy (i.e. no contraindications) and 129 will be ineligible and undergo alternative treatments. We estimate that approximately 20% will be lost to follow-up at the twelve-month visit, so that we will have complete data on a total of 922 women (57.5% of 1,602) treated with either CO_2_-based cryotherapy, LMIC-adapted CryoPen^®^, or thermoablation. We will monitor the percentages of ineligibles, contraindications, and loss to follow-up in real time and adjust recruitment accordingly to achieve the target sample size. In addition, we will conduct a non-essential 6-week follow-up visit to evaluate adverse effects from the ablative procedures. The primary endpoint of the study will be to determine residual disease at a 12-month follow up visit as determined by colposcopy and four-quadrant biopsies.

**Enrollment and Treatment**

Enrollment diagnosis will be based on the local pathology. These slides will be read by an expert gynecologic pathologist for quality assurance. At the pre-treatment visit, consented women will be administered a short questionnaire to collect demographic information and health history. Women will receive a pregnancy test as part of the eligibility criteria for enrollment in the study. Vaginal samples will be collected to be analyzed for bacterial vaginosis, yeast, gonorrhea, trichomonas, and chlamydia. Samples will be collected again at the 6-week follow-up visit to conduct the same analyses. Pathology samples from Latin America will be sent to the Pathology Department at the Medical College of Wisconsin (Milwaukee, WI) for molecular analyses. The China samples will be analyzed in-country at both SDH and Hybribrio labs using molecular tests as well. In addition, the ability to pass the cervical swab used for vaginal samples into the endocervix will be recorded at both the pre-treatment and the six-week follow-up visits to gather information about treatment-induced stenosis. All women will also undergo HPV genotyping with next-generation sequencing. The specimen is taken prior to treatment to establish a baseline of which HPV types are present. If a different HPV type is present in the post-treatment specimen, this will be classified as a new rather than persistent infection. Evaluation of the HPV genotyping from Latin America will be conducted at the National Cancer Institute in the United States and in China, where these samples will be analyzed at local labs. All samples that are sent to the United States for analysis will be shipped using a specialized courier service.

All women will also undergo a pelvic exam and visual inspection of the cervix following placement of diluted acetic acid on the cervix. Women will be immediately withdrawn from the study if any of the following contraindications for cryotherapy are present on visual inspection 1) lesion extends to over 75% of the cervix, 2) lesion is too large to be covered by the cryotherapy probe, 3) lesion extends into the endocervical canal, 4) the cervix is distorted or has severe atrophy; 5) the cervix is not visible, 6) the squamo-columnar junction is not completely visible, 7) the cervix is hard to reach, and/or 8) suspicion of cancer. Women deemed ineligible for cryotherapy will undergo LEEP immediately. In the case of suspected cancer, women will be appropriately referred. Once a patient is deemed eligible for cryotherapy, the study coordinator at each site will log into REDCap (a secure, HIPAA-compliant web application for building and managing surveys and databases) to obtain the randomized treatment assignment.

Women will then undergo treatment with the LMIC-adapted CryoPen^®^, the WISAP thermoablator, or CO_2_-based cryotherapy. Patient pain will be evaluated four times during the treatment visit as described in section 5.2 below.

Images of the cervix will be taken with the EVA System Mobile ODT device before and after treatment. De-identified cervical images from the initial visit and the follow-ups will be made available to Mobile ODT. The company will use these images to help develop an Automated Evaluated System (AVE), a software algorithm to detect pre-cancerous lesions through images rather than laboratory tests. This algorithm is currently in development and the more images it “learns”, the more accurate results it can obtain. If successful, AVE can greatly ease diagnosis and triage in low-income settings.

**Six-week follow-up**

Six weeks post-treatment, women will return for a visit to evaluate side-effects. Data will be collected on women who attend this visit at any time between 4 to 12 weeks post-treatment. If a woman does not attend this visit, she will still remain in the study and be invited to return at 12 months post-treatment. At the 6-week visit, women will complete a questionnaire to assess the presence of pain or cramps, bleeding, watery discharge, and malodorous discharge. Women will also be asked some questions about their attitudes and perceived acceptability of the treatment they received. Finally, women will be asked to answer a checklist to assess their compliance with post-treatment recommendations. In addition, a pelvic exam will be performed and vaginal samples will be collected and analyzed for bacterial vaginosis, yeast, gonorrhea, trichomonas, and chlamydia. If an infection is suspected, the provider may prescribe medication as necessary. The provider will note whether a vaginal culture swab can be introduced into the endocervix. If it cannot, stenosis will be diagnosed. Women will also be evaluated for acute clinical cervicitis at the follow-up visit and this will be recorded. Another photo will be taken with the Mobile ODT device.

**12-month follow-up**

One year post-treatment, women will return for a follow-up visit to determine residual disease, which is the primary endpoint. Residual disease will be determined by colposcopy and four-quadrant biopsies. In addition, VIA, cytology, and high-risk HPV DNA testing with careHPV will be performed. Genotyping and next-generation sequencing will be repeated to differentiate between persistent and new infections. The goal is to evaluate which test has the highest sensitivity after an ablative treatment: VIA, cytology, or HPV.

We will use local health systems, including healthcare promoters, to encourage women to return for the follow-up visit. Attempts to contact women who do not return for this visit will continue until 18 months post-treatment. If a woman does not return within this time period, she will be considered lost to follow-up.

At the 12-month visit, the following procedures will be performed:

1. Pelvic examination
2. Urine pregnancy test
3. Unaided VIA
4. Cytology
5. High-risk HPV testing with careHPV
6. Colposcopic evaluation and impression
7. Four-quadrant microbiopsy protocol using the protocol for sensitive detection of any recurrent/untreated CIN2+.^11^ All visible lesions in each quadrant of the cervix (12-3, 3-6, 6-9, and 9-12 o’clock) will be biopsied. Endocervical curettage (ECC) will be performed on all patients. If no lesion is visible in a given quadrant, the most suspicious appearing area in that quadrant will be biopsied.
8. Cervical swab collection for HPV genotyping using next-generation sequencing (NGS)

Treatment acceptability questions will again be administered at the end of this visit. Women diagnosed with recurrent/untreated CIN2+ on biopsy will be asked to return to undergo LEEP. Women with suspected cancer on biopsy will be referred to the local cancer hospital for standard of care.

### 3.2 Sample size and statistical analyses

**Treatment efficacy**

*Analytic Approach*. The primary analysis will follow an “intent to treat” approach, and we will conduct an additional sub-analysis excluding patients referred to excisional treatments after randomization. Similarity of the two study arms in terms of follow-up rates, distribution of individual and demographic variables, and disease distributions will be assessed. We will calculate the cure fraction and the corresponding binomial 95% confidence interval (95% CI) of each treatment as the percentage of women diagnosed with CIN2+ at the twelve-month visit. The non-inferiority of the LMIC-adapted CryoPen^®^ and the WISAP thermoablator relative to CO_2_-based cryotherapy will be assessed by calculating 95% CIs for the differences (LMIC-adapted CryoPen^®^ minus CO_2_-based cryotherapy and WISAP thermoablator minus CO_2_-based cryotherapy) in cure fractions. Non-inferiority will be concluded if the lower confidence bound is greater than the non-inferiority margin (-10%). Exploratory analysis of the effectiveness and differences between arms will be evaluated within strata defined by quartiles of age and by the baseline diagnosis. Both the local and expert pathologists’ diagnoses will be used in these analyses. As warranted, a logistic regression model will be used to mutually control for all important variables and chance differences between study arms to identify the key determinants of treatment failure and differences (if any) in effectiveness between treatments.

*Statistical Power and Sample Size*. With a total of 922 women with a diagnosis of CIN2+, 307 per treatment arm, the study will have at least 80% power (alpha = 0.05) to detect a non-inferiority margin difference of 10% between the cure fraction of CO_2_-based cryotherapy (reference) and of the LMIC-adapted CryoPen^®^ (experimental) and the WISAP thermoablator (experimental), for a range of efficacy (50% to 80%) for the reference treatment and 40% efficacy under the null hypothesis of inferiority.

We chose a range of 50-80% efficacy for cryotherapy because although the literature suggests CO_2_-based cryotherapy is 80% effective, the disease ascertainment by colposcopy following treatment in previous studies was before the recognition that colposcopy is not perfectly sensitive. Therefore, the disease ascertainment in those studies was not as rigorous as proposed in this study. To ensure that the study is sufficiently powered for a wide range of efficacies, we are selecting 50% efficacy for the control arm as the worst-case scenario for study power. In order to achieve a sample size of 922 with complete follow-up, we will enroll 1281 women with CIN2+ under the assumption that 10% will have contraindications for cryotherapy and 20% will be lost to follow-up at twelve months post-treatment.

**Test of cure**

*Analytic Approach*. Since all women returning for the twelve-month visit will undergo four-quadrant biopsies and LEEP if diagnosed with CIN2+, regardless of the baseline characteristics or ablative treatment, we can assess the test of cure performance (sensitivity, specificity, positive predictive values (PPV), and negative predictive values (NPV)) of each test (VIA, cytology, and high-risk HPV DNA) for recurrent CIN2+ and CIN3 diagnosed by local and expert pathology. Performance will be stratified by quartiles of age and by the baseline diagnosis. McNemar chi-square will be used to test whether differences in sensitivity and specificity are significant (p < 0.05). A method developed by Leisenring *et al.*^12^ will be used to test for significant differences in PPV and NPV.

*Statistical Power*. The statistical power for comparing sensitivity for the test of cure for VIA, cytology, and high-risk HPV testing is dependent on sample size of the efficacy study and the actual efficacy. We are enrolling 1281 women to assure that we have 930 women with a twelve-month follow-up. The larger the number of women who return for their follow-up, the larger the sample size for a given level of efficacy will be. The more efficacious the treatments, the smaller the sample size will be. Assuming 70% efficacy in treatment (30% recurrence), results of tests for cure will be available for 279 women with recurrence. This will provide 85% power (0.05 two-sided alpha) to detect a 10% difference in sensitivity between tests assuming 20% discordance between tests.

*Interim Analysis of Cure Fractions*. An interim analysis of cure fractions will be performed after approximately 2 years of study opening, when approximately 1/3 of the women have been enrolled. A confidence interval will be calculated for the difference (LMIC-adapted CryoPen^®^ minus CO_2_-based cryotherapy and WISAP thermoablator minus CO_2_-based cryotherapy) in cure fractions. If the upper bound of the confidence interval is lower than the non-inferiority margin (-10%), then treatment modification or termination may be considered. The confidence interval will be calculated using an alpha calculated using an alpha time-squared spending function to maintain an overall 5% Type I error (one-sided).

# 4.0 Research participants

### 4.1 Characteristics of research participants

The study will enroll eligible, consenting women aged 18 and older. Eligible women will be informed about the study and invited to participate. The legal age of consent in El Salvador, Peru, Colombia and China is 18 years of age.

### 4.2 Inclusion and exclusion criteria

Women who present to the health facility for biopsy results and meet the study criteria will be invited to participate in the study. We will collect delinked, anonymous, pooled data (e.g., age, geographic area of residence, and diagnosis) on all women, including those who decline to participate, to characterize them and assess whether our study population is representative of the general population.

**Criteria for Inclusion**

- Women aged 18 and older
- Biopsy results of CIN2, CIN2-3, CIN3, or high-grade CIN not otherwise specified (quality assurance will be performed by an expert gynecologic pathologist)
- Willing and able to provide informed consent
- Willing and able to provide permanent or reliable address

**Criteria for Exclusion**

- Pregnant or plans to become pregnant during study
- History of total hysterectomy (verified by medical record or pelvic evaluation)
- Previous surgery destructive to the cervix within the last 5 years
- Patient not eligible for cryotherapy (1) lesion extends to over 75% of the cervix, 2) lesion is too large to be covered by the cryotherapy probe, 3) lesion extends into the endocervical canal, 4) the cervix is distorted or has severe atrophy; 5) the cervix is not visible, 6) the squamo-columnar junction is not completely visible, 7) the cervix is hard to reach, and/or 8) suspicion of cancer)
- Cervix shape disfigured or hard to reach

### 4.3 Withdrawal from the study

Women who sign the informed consent to participate in the study have the right to withdraw their consent at any time during the study. Physicians performing the ablation procedures have the right to remove participants from the study if, upon pelvic examination, the condition of the cervix is not conducive to continuing the procedure.

# 5.0 Study procedures

### 5.1 Initial Exam

After a woman consents to participate in the research study and meets other eligibility criteria including testing negative on a pregnancy test, she will answer a short survey with demographic and life history information relevant to the study (i.e., age, location of residence relative to clinic, smoking and sexual history, parity and history of vaginal surgeries). Next, the provider will initiate the ablation procedure by performing a pelvic exam. If the provider determines during this exam that the condition of the cervix is not conducive to an ablation procedure for anatomical reasons, the participant will be removed from the study.

### 5.2 Study procedures for cervical ablations

For the traditional cryotherapy arm, providers will use a MedGyn cryotherapy unit with a 19 or 25 mm conical tip supplied with a refillable tank of medical grade CO_2_. For the experimental CryoPen^®^ arm, providers will use LMIC-adapted CryoPen^®^ prototype with a 19 mm conical tip. For the thermoablator arm, providers will use a handheld WISAP thermoablator (model C3) with a 20 mm conical tip.

Anesthesia is not required for cervical ablation procedures and none will be provided routinely in this research study. Analgesics may be prescribed for discomfort after the procedure. Study coordinators will verbally ask study participants to assess their pain using the Wong Baker Faces Scale (Figure 2),^13^ a well-validated and widely used self-assessment tool. This scale was originally developed for use with children but is now available in many languages, including Spanish and Mandarin Chinese, and is used to improve communication between patients and providers. It utilizes five simple drawings of faces depicting different levels of pain, each shown above a number from 0 (no pain) to 10 (worst possible pain). Patients can use the expressions on the faces to rate their own experience according to the corresponding number. Pain will be assessed at 4 points: before speculum insertion (baseline), after speculum insertion but before treatment, during treatment, and after speculum removal.


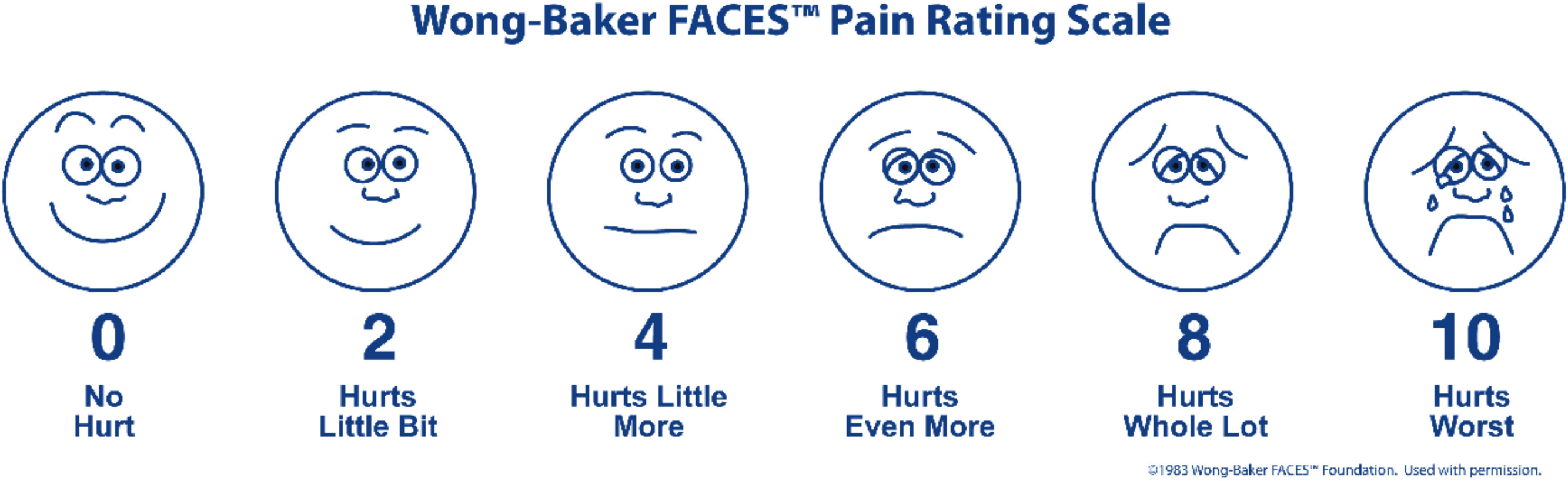


Figure 2. FACES and numeric pain rating scale

Cervical photographs will be taken immediately pre- and post-procedure for participants who have consented to photographs. Photographs will provide additional documentation of the cervix anatomy to corroborate the clinical evaluation whether the anatomy of the cervix could have interfered with a good contact by the treatment probe at cervix positions 3, 6, 9 or 12 (as on a clock face).

Each study procedure is estimated to take approximately 10-15 minutes. The remainder of the time will be spent preparing the participant and taking cervical photographs.

The six-week and 12-month follow-up visits (described in section 3.1) are expected to take about 15 minutes. All women will undergo liquid-based cytology, VIA, and HPV testing. Women suspected of having invasive cancer at the follow-up visit will be referred for appropriate management.

**5.2.1 CO_2_-based cryotherapy procedure**

- Study physicians will use MedGyn cryotherapy units supplied with a refillable tank of medical grade CO_2_ and a 19 or 25 mm conical tip.
- A pressure gauge attached to the compressed gas tank shows the cylinder gas pressure. If there is adequate gas pressure in the cylinder (40-74 kg/cm^2^), the indicator moves to the green zone in the gauge when the cryogun releases the gas. The pressure must be in the green zone or there will be insufficient freezing for cryotherapy.
- The cervix is exposed after a speculum is placed to separate the vaginal walls.
- The cryotherapy probe tip is applied with the center of the tip on the external os. The probe should adequately cover the lesion and should not come in contact with any part of the vagina during the procedure.
- The gas trigger in the cryogun is squeezed to administer the freeze. An ice ball will form on the tip of the cryoprobe and on the cervix throughout the freeze.
- A double freeze is applied.
- The cryoprobe is removed by gently rotating on the cervix once complete thawing has occurred and the ice ball has cleared.
- The cryoprobe and tip must be disinfected using a high-level disinfectant.

**5.2.2 CryoPen^®^ procedure**

- Study physicians will use the LMIC-adapted CryoPen^®^ with a 19 mm conical tip.
- The CryoPen unit is plugged into an electrical outlet. The well is filled with ethanol. The unit is ready for use when the light on the temperature indicator turns green.
- The tip is placed on the applicator and twisted in place.
- The core is removed from the unit and the well is closed to prevent the well from freezing.
- The core is inserted into the applicator and pulled against the core handle to hold the device together.
- The core and the applicator are inserted together into the vagina and the tip is placed on the cervix.
- When the procedure is completed the core is removed first. Once complete thawing has occurred, the applicator is then removed by gently rotating the tip.
- If the entire SCJ is not frozen, providers may repeat the application once more until the entire SCJ is ablated.
- The core is then returned to the CryoPen^®^ unit. Frost must be wiped from the core with a towel before the core is inserted into the well.
- The applicator must be discarded and the core tip must be disinfected using a high-level disinfectant.

**5.2.3 Thermoablator**

- Study physicians will use a handheld thermoablator with a 20 mm conical tip, manufactured and sold by WISAP Medical Technology (Germany).
- The probe and protector slider are connected to the unit and the unit is plugged into an electrical outlet.
- The unit is turned on when the power button is pressed to the “on” position. The switch illuminates green and starts blinking as the therapy probe tip begins to heat up.
- Unit is ready for use when the green temperature indicator light stops blinking.
- The physician slides the protector forward to cover the probe tip.
- When the probe makes contact with the cervix, the protector is moved back.
- The probe tip is applied to the cervix for 40 seconds.
- The protector is slid back to cover the tip.
- The probe is removed and the physician visually evaluates the cervix to ensure the entire SCJ has been ablated.
- In some cases, providers may continue with additional 20 second applications until the entire SCJ is ablated.
- When the procedure is completed the unit is turned off and the probe and protector are removed for disinfection with a high-level disinfectant.

5.3 Laboratory evaluation

A local pathologist will evaluate initial and follow-up cervical biopsy specimens. Enrollment diagnosis will be based on the local pathology. These slides will also be reviewed by an expert gynecological pathologist for quality assurance. At the one year follow-up visit, women will have colposcopic-directed biopsies. This pathology will be read by both a local and an expert gynecologic pathologist. The primary endpoint of the study will be the consensus achieved by the local and expert pathologists on the follow-up biopsy samples taken from the colposcopic-directed biopsies at the one year follow up visit. Discrepant cases will be discussed between the two pathologists and if agreement cannot be reached, the case will be reviewed by a third expert pathologist. Consensus will be defined as two of the three pathologists agreeing on the diagnosis. We hope that by using this consensus method, the true incident of CIN2+ will be able to be evaluated and bias in favor of superior cure rates will be avoided. Standard cytology will be taken as per local guidelines. Cytology will be read by a local pathologist and interpreted according to the Bethesda System for classification.^19^ Evaluation of the HPV genotyping will be conducted at the National Cancer Institute.

# 6.0 Recruitment

Hospital clinicians at Salvadorian Social Security Hospital (ISSS) in San Salvador, El Salvador, La Liga Contra el Cáncer in Lima, Peru, Hospital Universitario San Ignacio (HUSI), Ginesalud SAS in Bogota, Colombia and the four hospitals in China (Shanxi Shanxi Day Hospital, Yanjin County Women and Children Hospital, Fengqiu County Women and Children Hospital, and Weihui Municipal Hospital for Women and Children Healthcare) will be informed of study criteria and will alert local study coordinators when participants with CIN2+ are scheduled for procedures.

- Our collaborators will be paid per patient during the course of this study. To help ensure that all of the data is obtained, the remainder of the compensation will be provided after the follow-up visit is completed.
- Potential participants will be invited by hospital staff to a private room to learn about the study. This conversation is anticipated to last approximately 30 minutes. The potential participant’s family members will be invited to join this conversation if the woman so chooses.
- The study coordinator will assess inclusion/exclusion criteria to confirm eligibility based on the potential participant’s clinical record of the most recent pelvic exam, performed either prior to or after her hospitalization, and invite her to participate.
- Participants will be informed that only information necessary to confirm eligibility will be entered into the study data collection form. This form will have a number code for the patient, not the participant’s name.
- As part of the information provided, women in El Salvador, Colombia, and Peru will be informed that their tissue sample will be analyzed in a laboratory in the United States. All women in the study will be informed that results of their analysis and their de-identified information may be published in a peer-reviewed medical journal. Women will be informed that no personal information will be in any analysis of results or publication.
- As part of the information provided, women will be informed that photographs may be taken of the uterine cervix during their procedures. The photographs will be assigned a study number and will not identify the participant. Women may opt out of having photographs taken. De-identified images will be made available to Mobile ODT to develop an algorithm to screen pre-cancerous lesions.
- Women will have the opportunity to ask the study coordinator questions about the study of the study coordinator. In addition, the study coordinator will ask the potential subject to repeat the major points of the study in order to ensure that the woman understands to what she is agreeing and what is expected of her.
- Women who decline to participate will be offered the standard of care treatment for CIN2+.
- The study coordinator will not be the primary doctor for the potential study participants and will not be in a relationship of authority over participants. The study coordinator will explain that the woman’s decision to participate in the study will not influence her care.

# 7.0 Consent process

- The study coordinator, the Principal Investigator (PI), and the co-investigators all have certificates showing completion of training in human subjects protections within the last five years.
- Consent will occur at the same time as recruitment by the study coordinator, which is described above. As stated above, a potential participant will be in a private room. The woman’s family members will be invited to join this conversation if the woman chooses.
- Women will be invited to participate in the study at the time they receive their diagnosis of CIN2+.
- It will be made clear to women that the research study is not a necessary part of their care.
- A woman who agrees to participate in the study will be provided an informed consent form. All women who agree to participate will sign the form or provide a fingerprint if non-literate. A witness will be present.
- If any non-literate women are invited to participate in the study, they may sign the consent form with a thumbprint. The witness will sign the consent form acknowledging that the woman understands the study.
- Women who consent to participate in the study will be randomized to a study arm immediately prior to her ablation
- Women who sign the informed consent to participate in the study have the right to withdraw their consent at any time during the study and stop their participation in the study without affecting their treatment.

# 8.0 Study instruments

At the beginning of the research study, a meeting of all staff involved in the study will be held. The protocol and all procedures for the study will be presented and discussed. The discussion will include use of the instruments and their safe storage and handling. The operating instructions or manuals for all three instruments will be provided at the meeting.

Providers in this study have been trained previously in all aspects of the handling and use of the standard cryotherapy equipment, the adapted CryoPen^®^, and the WISAP thermoablator model C3. Any providers who have not yet used the CryoPen^®^ or thermoablator will be trained in safe handling, use, sterilization, and storage by a CryoPen, Inc., or WISAP employee or the PI of this study, Dr. Miriam Cremer.

All instruments used in this study are the property of the Cleveland Clinic. The devices may be donated to the institutions where they are being used at the end of the study. Safe use, storage, and disposal is the responsibility of the institution. Specifically, each institution is responsible for ensuring that the use of all instruments conform to regulatory approval granted for each, whether for clinical or research use.

The MedGyn cryotherapy instrument is approved and widely used around the world. The CryoPen^®^ freezes tissue in the same manner as the MedGyn instrument: a metal probe cooled to -50ºC to -80ºC is applied to the cervix. The WiSAP thermoablator (model C3) ablates tissue with heat by applying a probe heated to 100ºC to the cervix.

# 9.0 Risks

Traditional cryotherapy is a safe and effective treatment for precancerous cervical lesions. The CryoPen^®^ is a recently developed instrument and has FDA approval for gynecological procedures. It freezes tissue in a manner similar to the traditional method and side effects are similar. In undergoing procedures in this study, women may experience some discomfort related to the insertion of the speculum. Some pain, even severe pain, and cramping may occur during freezing, but these effects are minor and short-lived. While pain evaluations are very subjective, we expect patients’ pain levels to allow them to leave the hospital or clinic on their own. Some women may experience a vasovagal reaction during treatment, but this is expected to resolve quickly after treatment is completed. Women infrequently experience discharge, bleeding, and/or cramping within one month of traditional treatment that causes them to seek medical care, and long-term sequelae, such as stenosis and a negative impact on fertility and obstetrical outcomes, appear to be uncommon. Vaginal infections post-treatment may occur but are infrequent. Severe bleeding during and/or after traditional cryotherapy treatment requiring further medical attention or blood transfusion is an extremely rare occurrence. The development of pelvic inflammatory disease (PID) following cryotherapy is rare. Definitions and reporting guidelines of adverse events can be found in Section 13.

Since clinicians at the study sites in El Salvador (Salvadorian Social Security Hospital), Peru (La Liga Contra el Cáncer in Lima), Colombia (Hospital Universitario San Ignacio and Ginesalud SAS), and China (Shanxi Dayi Hospital, Yanjin County Women and Children Hospital, Fengqiu County Women and Children Hospital, and Weihui Municipal Hospital for Women and Children Healthcare) are experienced gynecologists, no additional risk than would normally be expected for this procedure is anticipated. No additional risks are expected for CryoPen^®^ since the amount of tissue damage is expected to be similar to that achieved with conventional cryotherapy. Women receiving a procedure will be provided information about managing the immediate minor symptoms.

Reviews of cold coagulation performed for more than 2500 women in Scotland reported that they occasionally experienced cramps but that anesthesia was not required in 95 percent of patients.^11,12^ Bleeding occurred rarely, and this was usually from biopsy performed before treatment. The heat of the probe causes blood to clot, reducing the risk of bleeding from the procedure itself. As with freezing, there may be some pain, including severe pain, during the procedure, and cramping, discharge, and/or bleeding after the procedure and a risk of infection post-procedure. As with cryotherapy, we expect pain levels to subside quickly after treatment and allow women to leave on their own.

All women will be evaluated 6 weeks after treatment. The data from this visit will be evaluated in the bi-monthly research calls. We expect that the side effects from all ablative treatment will be minor, however, if there are any unanticipated adverse events, serious adverse events, or other unanticipated problems these will be discussed as a group and we will decide whether to continue with that arm of the study.

There is also the risk that the ablative procedure will not treat the underlying CIN2+ lesion. According to a Cochrane review, cure rates for cervical intraepithelial neoplasia grade 3 (CIN3) following cryotherapy range from 77% to 93%^20^. Women who participate in the study will all undergo colposcopy and 4-quadrant biopsy at 12 months. Thus, residual disease will be identified and women who still have CIN2+ will be referred for treatment with LEEP.

# 10.0 Mitigation of risks

If a participant is experiencing significant pain during the procedure she may request that the procedure be stopped. After the procedure, participants will be offered analgesics such as acetaminophen or other medication as deemed appropriate by her care team. In the unlikely event that a complication occurs, the local study staff have the resources available at these high-level hospitals to assist the subject and provide appropriate clinical care for any condition. To mitigate side-effects and the risk of infection, women will be provided with standardized post-treatment recommendations that include avoiding the following behaviors: sexual intercourse, vaginal douches, bathing or swimming in any body of water, and the insertion of tampons or other objects into the vagina.

All women are scheduled to have two follow-up visits— 6 weeks and 12 months post-treatment. The risk of an untreated lesion developing into cancer during this time is extremely low^21-23^. Several attempts will be made to contact women to be sure that they come in for follow-up.

# 11.0 Benefits

Patients will receive treatment of cervical precancer. They will also have a higher level of post-operative surveillance (VIA, cytology, and HPV testing) than women who do not participate in the study. Furthermore, women will have quality analysis done on all of their pathology specimens.

# 12.0 Study and safety monitoring

The PI and the study coordinators are staff members of the institutions participating in the study; general study supervision will be conducted by the PI. The local study coordinators will conduct recruitment, consent, and interviews of potential study participants. Sample labeling will be conducted by the study coordinators. The patient database will be managed by the research staff secretary and/or study coordinators. The PI and study coordinators will conduct adverse events monitoring.

***Data and Safety Monitoring Plan***. Participants will include the principal investigator and study co-investigators, in addition to an epidemiologist, an OB-GYN, a gynecologic oncologist, and 2 researchers who are not part of the study. After enrollment begins, the group will meet bi-monthly by telephone conference and additionally if any unanticipated Adverse Events (AEs), Serious Adverse Events (SAEs), or Unanticipated Problems (UPs) occurs. They will assess enrollment, loss to follow-up, any safety issues or concerns including pain due to treatment, and perform an interim analysis.

***Interim Analysis of Cure Fractions.*** An interim analysis of cure fractions will be performed after two years from study opening when approximately 1/3 of the women have been enrolled. Confidence intervals will be calculated for the differences (LMIC-adapted CryoPen^®^ minus cryotherapy and WISAP thermoablator minus cryotherapy) in cure fractions. If the upper bound of the confidence interval is lower than the non-inferiority margin (-10%), then treatment modification or termination may be considered. The confidence interval will be calculated using an alpha calculated using an alpha time-squared spending function to maintain an overall 5% Type I error (one-sided).

# 13.0 Managing and reporting adverse events and unanticipated problems

### 13.1 Adverse Events and Unanticipated Problems

An **adverse event (AE)** is any untoward or undesirable event experienced by a participant regardless of whether the event is expected or related to the participant’s involvement in the research.

An **unanticipated problem (UP)** is an unexpected event given the research procedures that are described in the protocol, and given the characteristics of the population being studied; it is related or possible related to participation in the research; and it suggests that the research places subjects or others at a greater risk of harm (including physical, psychological, economic, or social) than was previously known or recognized.

### 13.2 Serious Adverse Events

A serious adverse event (SAE) is any untoward medical occurrence or effect that involves

- Death
- A life-threatening condition (participant at immediate risk of death)
- Persistent or significant disability/incapacity
- In-patient hospitalization or prolongation of existing hospitalization
- A congenital anomaly or birth defect
- An important medical event that may jeopardize the participant or may require intervention to prevent one of the outcomes listed above.

AEs, SAEs, and unanticipated problems will be reviewed and evaluated by the site PIs throughout the study. They will decide how to report these according to the guidelines below.

All AEs, SAEs, and unanticipated problems will be followed until satisfactory resolution or until the PI deems the event to be chronic or the patient to be stable. Qualified health providers will provide treatment of study-related adverse events at the study sites. Treatments administered are to be recorded on the participant’s data collection form as a complication and will be reported as necessary to the local and Cleveland Clinic’s Institutional Review Boards (IRBs).

### 13.3 Reporting procedures for AEs, SAEs, and unanticipated problems

The study coordinator at each study site will notify the principal investigator of (Dr. Miriam Cremer) of every AE that meets the criteria for an unanticipated problem, SAE, and/or other unanticipated problem within 24 hours of finding out about the event, even if the study coordinator considers the event not related to the study or is unsure about relatedness to the study.

The PI will report AEs that meet the criteria for unanticipated problems, SAEs, and other unanticipated problems to the Cleveland Clinic IRB according to REC guidelines. Every unanticipated AE, SAE, and/or unanticipated problem occurring during the study must also be reported to the local IRB in accordance with local reporting requirements.

Initial notification of the unanticipated AE, SAE or unanticipated problem to the study PI either via phone, fax, or email must be made within 24 hours of the site becoming aware of the event. The initial notification should be coded as “Initial AE/SAE notification” and should include as much of the information on the AE/SAE reporting form as possible including a description and date/s of onset.

The PI should not wait for additional information to fully document the event before notification to Cleveland Clinic. The initial notification is to be followed by a completed adverse report form as soon as possible, but at latest within three calendar days of the initial notification. The completed adverse form should be coded as “Follow-up Adverse Event report” or “Final Adverse Event report” depending on whether the event is ongoing or has been satisfactorily resolved, and should detail relevant aspects of the adverse event/s, action/s taken and event outcomes. If applicable, hospital case records and autopsy reports should be obtained.

Any relevant information that becomes available after the SAE report form has been sent (outcome, precise description of medical history, results of the investigation, copy of the hospitalization report) should be forwarded as soon as possible to the study PI. The confidentiality of the participants shall be respected when forwarding this information.

# 14.0 Confidentiality and data management

All study documents will be coded and this information will be kept in a locked storage room in the research office in the respective countries. Copies will be electronically sent through a secure website to the PI and the study coordinator in the US.

Coded data will be stored in REDCap database. Data that is sent from El Salvador, Peru, Colombia and China to the United States via email will be sent in encrypted form.

Any linking documents with the study identification number and women’s names will be securely stored and kept separately from the other study records. These linking documents will also be entered into a separate, password-protected and HIPAA-compliant electronic storage folder or file. Only essential biological samples and photographs of the cervix will be labeled with the patient study code and will not include any identifiers to the subject. Following the standard procedures at for any biopsy, tissue will be stored in secure facilities for ten years before being destroyed. No further research will be performed on this tissue.

Study staff or clinicians who are obligated to follow the participant for clinical care reasons will have access to this linking document in paper or electronic form. Linking documents will be destroyed as soon as the study is completed.

Research ethics committees with oversight for this study will have the right to review research data. A report of study results will be created and shared with the cervical cancer community. No individual patient identifiers will be included. Manuscripts for journal submission may be prepared.

# 15.0 Study costs

The costs of the study are borne by the sponsor. There is no cost to the patient for participating in the study.

# 16.0 Care for injury

Since cervical ablation procedures are common and are not high risk, and the physicians at the study sites are experienced specialists, no injuries are anticipated. In the unlikely event of injury appropriate care will be provided at all sites. All subjects are undergoing treatment for their high-grade lesion with ablative therapy, which is the standard of care. Participants are responsible to follow up at six weeks and one year to ensure that their lesions have been treated.

# 17.0 Compensation

In El Salvador, Peru, Colombia and China it is unusual to offer compensation or payment for participating in a research study because this adds pressure to participate, so no payment will be offered.

# 18.0 Investigator responsibilities

Dr. Miriam Cremer, Associate Professor at Cleveland Clinic and Founder & President of Basic Health International, is the Principal Investigator.

**Site Principal Investigators include:**

Ana Celia de Uriarte, M.D. – El Salvador (ISSS)

Henry Valdivia Franco, M.D. – Peru (La Liga)

Raul Hernando Murillo Moreno, M.D. – Colombia (HUSI)

Gustavo Ciendua, M.D. Sonia Salamanca, M.D. and Diana Valderrama, M.D. – Colombia (Ginesalud SAS)

Suhui Wu, M.D. – China (SDH)

All PIs will submit the initial paperwork and IRB protocol the local IRBs. Dr. Cremer will submit the IRB to the Cleveland clinic. General study coordinators will be responsible for creating and managing the randomization list. The local study coordinators will conduct recruitment, consent, and interviews of potential study participants. Sample labeling will be conducted by the local study coordinators. Specimen processing will be conducted by study pathologists. The patient database will be managed by general study coordinators. Adverse events monitoring will be conducted by the PI and the study coordinators. Study supervision will be conducted by the PI. Reporting of data will be conducted by the PI and the study coordinators. Data analysis, writing, and publication will be conducted by all collaborators.

References

1. Ferlay J, Shin HR, Bray F, Forman D, Mathers C, Parkin DM. Estimates of worldwide burden of cancer in 2008: GLOBOCAN 2008. *International journal of cancer Journal international du cancer.* 2010;127(12):2893-2917.

2. Paavonen J, Naud P, Salmeron J, et al. Efficacy of human papillomavirus (HPV)-16/18 AS04-adjuvanted vaccine against cervical infection and precancer caused by oncogenic HPV types (PATRICIA): final analysis of a double-blind, randomised study in young women. *Lancet (London, England).* 2009;374(9686):301-314.

3. Group FIS. Quadrivalent vaccine against human papillomavirus to prevent high-grade cervical lesions. *The New England journal of medicine.* 2007;356(19):1915-1927.

4. Winer RL, Feng Q, Hughes JP, O'Reilly S, Kiviat NB, Koutsky LA. Risk of female human papillomavirus acquisition associated with first male sex partner. *The Journal of infectious diseases.* 2008;197(2):279-282.

5. World Health Organization. Global Status Report on Noncommunicable Diseases 2010. World Health Organization; 2011.

6. Santesso N, Schunemann H, Blumenthal P, et al. World Health Organization Guidelines: Use of cryotherapy for cervical intraepithelial neoplasia. *International journal of gynaecology and obstetrics: the official organ of the International Federation of Gynaecology and Obstetrics.* 2012;118(2):97-102.

7. Nene BM, Hiremath PS, Kane S, Fayette JM, Shastri SS, Sankaranarayanan R. Effectiveness, safety, and acceptability of cryotherapy by midwives for cervical intraepithelial neoplasia in Maharashtra, India. *International journal of gynaecology and obstetrics: the official organ of the International Federation of Gynaecology and Obstetrics.* 2008;103(3):232-236.

8. Sankaranarayanan R, Nene BM, Shastri SS, et al. HPV screening for cervical cancer in rural India. *The New England journal of medicine.* 2009;360(14):1385-1394.

9. Sherris J, Wittet S, Kleine A, et al. Evidence-based, alternative cervical cancer screening approaches in low-resource settings. *International perspectives on sexual and reproductive health.* 2009;35(3):147-154.

10. Sherris J, Wright TC, Denny L, et al. ALLIANCE FOR CERVICAL CANCER PREVENTION: SETTING THE RECORD STRAIGHT. *American Journal of Public Health.* 2007;97(2):200-201.

11. Pretorius RG, Zhang W-H, Belinson JL, et al. Colposcopically directed biopsy, random cervical biopsy, and endocervical curettage in the diagnosis of cervical intraepithelial neoplasia II or worse. *American Journal of Obstetrics & Gynecology.*191(2):430-434.

12. Leisenring W, Alono T, Pepe MS. Comparisons of Predictive Values of Binary Medical Diagnostic Tests for Paired Designs. *Biometrics.* 2000;56(2):345-351.

13. Wong DL, Baker CM. Pain in children: comparison of assessment scales. *Okla Nurse.* 1988;33(1):8.

14. Bergeron C, Ordi J, Schmidt D, Trunk MJ, Keller T, Ridder R. Conjunctive p16INK4a Testing Significantly Increases Accuracy in Diagnosing High-Grade Cervical Intraepithelial Neoplasia. *American Journal of Clinical Pathology.* 2010;133(3):395-406.

15. Stoler MH, Schiffman M, Group ASCoUSL-gSILTS. Interobserver reproducibility of cervical cytologic and histologic interpretations: Realistic estimates from the ascus-lsil triage study. *JAMA.* 2001;285(11):1500-1505.

16. Galgano MT, Castle PE, Atkins KA, Brix WK, Nassau SR, Stoler MH. Using Biomarkers as Objective Standards in the Diagnosis of Cervical Biopsies. *The American Journal of Surgical Pathology.* 2010;34(8):1077-1087.

17. Dijkstra MG, Heideman DAM, de Roy SC, et al. p16INK4a immunostaining as an alternative to histology review for reliable grading of cervical intraepithelial lesions. *Journal of Clinical Pathology.* 2010;63(11):972-977.

18. Palma PD, Rossi PG, Collina G, et al. The Reproducibility of CIN Diagnoses Among Different Pathologists: Data From Histology Reviews From a Multicenter Randomized Study. *American Journal of Clinical Pathology.* 2009;132(1):125-132.

19. Solomon D, Davey D, Kurman R, et al. The 2001 bethesda system: Terminology for reporting results of cervical cytology. *JAMA.* 2002;287(16):2114-2119.

20. Martin-Hirsch PP, Paraskevaidis E, Bryant A, Dickinson HO. Surgery for cervical intraepithelial neoplasia. *The Cochrane database of systematic reviews.* 2013;12:CD001318.

21. Schiffman M, Wentzensen N, Wacholder S, Kinney W, Gage JC, Castle PE. Human Papillomavirus Testing in the Prevention of Cervical Cancer. *Journal of the National Cancer Institute.* 2011;103(5):368-383.

22. McCredie MRE, Sharples KJ, Paul C, et al. Natural history of cervical neoplasia and risk of invasive cancer in women with cervical intraepithelial neoplasia 3: a retrospective cohort study. *The Lancet Oncology.*9(5):425-434.

23. Schiffman M, Rodríguez AC. Heterogeneity in CIN3 diagnosis. *The Lancet Oncology.*9(5):404-406.
